# MPP^+^-Induced Changes in Cellular Impedance as a Measure for Organic Cation Transporter (SLC22A1-3) Activity and Inhibition

**DOI:** 10.3390/ijms23031203

**Published:** 2022-01-21

**Authors:** Tamara A. M. Mocking, Hubert J. Sijben, Yimé W. Vermeulen, Adriaan P. IJzerman, Laura H. Heitman

**Affiliations:** 1 Division of Drug Discovery and Safety, Leiden Academic Centre for Drug Research, Leiden University, Einsteinweg 55, 2333 CC Leiden, The Netherlands; t.a.m.mocking@lacdr.leidenuniv.nl (T.A.M.M.); h.j.sijben@lacdr.leidenuniv.nl (H.J.S.); y.w.vermeulen@umail.leidenuniv.nl (Y.W.V.); ijzerman@lacdr.leidenuniv.nl (A.P.I.); 2 Oncode Institute, 2333 CC Leiden, The Netherlands

**Keywords:** OCT, label-free, xCELLigence, MPP^+^, transport, SLC22A1-3

## Abstract

The organic cation transporters OCT1-3 (*SLC22A1-3*) facilitate the transport of cationic endo- and xenobiotics and are important mediators of drug distribution and elimination. Their polyspecific nature makes OCTs highly susceptible to drug–drug interactions (DDIs). Currently, screening of OCT inhibitors depends on uptake assays that require labeled substrates to detect transport activity. However, these uptake assays have several limitations. Hence, there is a need to develop novel assays to study OCT activity in a physiological relevant environment without the need to label the substrate. Here, a label-free impedance-based transport assay is established that detects OCT-mediated transport activity and inhibition utilizing the neurotoxin MPP^+^. Uptake of MPP^+^ by OCTs induced concentration-dependent changes in cellular impedance that were inhibited by decynium-22, corticosterone, and Tyrosine Kinase inhibitors. OCT-mediated MPP^+^ transport activity and inhibition were quantified on both OCT1-3 overexpressing cells and HeLa cells endogenously expressing OCT3. Moreover, the method presented here is a valuable tool to identify novel inhibitors and potential DDI partners for MPP^+^ transporting solute carrier proteins (SLCs) in general.

## 1. Introduction

To maintain cellular homeostasis, cells depend on transporters for the uptake and export of various solutes such as neurotransmitters, hormones, nutrients, and metabolites. Bidirectional transport of cationic endo- and xenobiotics across the cell membrane is facilitated by the organic cation transporters (OCTs) that belong to the SLC22 family. The three OCTs (OCT1-3, *SLC22A1-3*) are low-affinity, high-capacity transporters of neurotransmitters and other cationic molecules such as metabolites and drugs [1,2]. OCT1 and OCT2 are predominantly expressed in the liver and kidney, respectively, while OCT3 is expressed throughout the body [3]. The ability to transport and interact with structurally diverse molecules designates OCTs as regulators of drug disposition and excretion [4]. However, their lack of specificity and the myriad of cationic drugs on the market, e.g., beta-blockers, antihistamines, and antidepressants [5,6], make OCTs highly susceptible for drug-drug interactions (DDIs). DDIs could alter a drug’s pharmacokinetics and pharmacodynamics, and thereby hamper drug effectiveness or lead to adverse events [7].

Indeed, assessment of DDIs for novel drug candidates is highly recommended by the European Medicine Agency (EMA) and Food and Drug Administration (FDA) for OCT1, OCT2, and multidrug and toxic extrusion (MATE) transporters [5,8]. Hitherto, OCT3 was largely neglected in drug–drug interaction studies. Consequently, the pivotal role of OCT3 as primary transporter for doxorubicin uptake into cardiomyocytes was only recently discovered [9], even though doxorubicin-induced cardiac toxicity has been a serious adverse event for decades [10,11]. Inhibition of OCT3 was proposed as a therapeutic strategy to prevent cardiac injury by chemotherapeutics [9,12]. Unfortunately, OCT inhibitors that were identified to date fail to show subtype specificity, making selective targeting of OCTs challenging. Recently, several tyrosine kinase inhibitors (TKIs) were identified as OCT inhibitors, which could provide a starting point to improve selectivity between OCTs [13,14]. For example, the TKI nilotinib displays some selectivity towards OCT3 and could potently inhibit OCT3 activity in mice [9,14].

Current methodology to identify novel OCT inhibitors and preclinical in vitro evaluation of DDIs with OCTs relies on uptake assays utilizing labelled substrates in transiently overexpressing cells [7]. Labeling of substrates with radioisotopes or generation of fluorescent analogs is costly and labor intensive, while OCT inhibition is probe-dependent [15]. Therefore, label-free methodologies to study transporters are currently emerging as an alternative to traditional uptake assays [16]. Recently, our group introduced the xCELLigence-biosensor as a successful tool to evaluate transport activity of the equilibrative nucleoside transporter 1 (ENT1), dopamine (DAT) and norepinephrine (NET) transporters indirectly via attenuated G protein-coupled receptor (GPCR) signaling [17,18,19]. This impedance-based assay detects GPCR-induced changes in cell morphology, adhesion, and proliferation on intact cells [20] and provides a more physiologically relevant condition to assess transport activity than the conventional uptake assays without the need to label substrates. However, this ‘transport activity through receptor activation’ (TRACT) [17] assay requires coexpression of transporter and GPCR that share a common ligand/substrate.

In this study, a label-free, impedance-based transport assay is therefore introduced that measures OCT-mediated transport activity utilizing the neurotoxic substrate MPP^+^. MPP^+^-induced changes in cellular impedance were OCT-dependent and were already detectable within the first hour after substrate addition, in contrast to conventional cytotoxicity assays that usually take multiple hours to days [21,22]. Moreover, OCT3 activity and inhibition by OCT inhibitors was quantified on both OCT3 overexpressing and endogenously expressing cells. This assay is suitable for high throughput screening and could aid in early identification of potential DDI partners and in novel drug discovery campaigns.

## 2. Results

### 2.1. OCT3-Mediated Transport of MPP^+^ Induces a Peak Response in an Impedance-Based Cytotoxicity Assay

**Figure 1:**
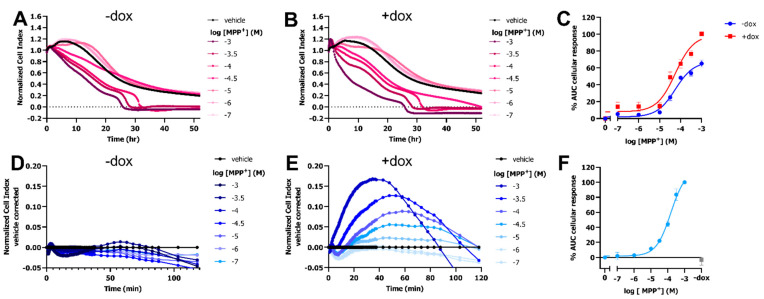
MPP^+^ uptake by cells expressing OCT3 results in a concentration-dependent increase in cellular impedance in first hour. Representative time-traces of dose-dependent MPP^+^ or PBS (vehicle)-induced response in HEK293-JI-OCT3 cells in absence (**A**) and presence (**B**) of doxycycline-induced OCT3 expression as measured for 52 h using xCELLigence RTCA system. Concentration-response curves of MPP^+^ on HEK293-JI-OCT3 cells pretreated with vehicle (−dox) or 1 µg/mL doxycycline (+dox) where area under curve (AUC) was obtained over 52 h (**C**) after vehicle-correction (Figure S1). 1 mM MPP^+^ response in presence of doxycycline was set at 100% (**C**). Representative vehicle-corrected time-traces of dose-dependent MPP^+^ or PBS (vehicle) induced response in HEK293-JI-OCT3 cells in absence (**D**) and presence (**E**) of doxycycline-induced OCT3 expression as measured over first 2 h. Concentration-response curve of MPP^+^ on HEK-JI-OCT3 cells pretreated with vehicle (−dox) or 1 µg/mL doxycycline (+dox) where AUC was obtained over first 60 min and 1 mM MPP^+^ response in presence of doxycycline was set at 100% (**F**). Gray bar represents 1 mM MPP^+^-induced response on vehicle treated cells (**F**). Data shown are mean ± SEM of at least three independent experiments performed in duplicate.

To investigate if OCT-mediated uptake of the neurotoxic substrate MPP^+^ led to distinct changes in cellular impedance, a HEK293-JumpIn cell-line was used with doxycycline (dox)-inducible OCT3 expression (HEK293-JI-OCT3). A stable cellular impedance (CI) was obtained after culturing the vehicle- and dox-induced HEK293-JI-OCT3 cells in a so-called E-plate for 22–24 h to reach confluency. This basal impedance enabled cytotoxic evaluation of MPP^+^ on cells in absence (-dox) and presence (+dox) of OCT3 expression using the xCELLigence real-time cell analyzer (RTCA) (Figure 1A,B and Figure S1). Total loss of cellular impedance was observed for cells treated with >100 µM MPP^+^ within 24 to 32 h (Figure 1A,B), observed as a reduction in normalized CI (nCI) over time. Both induced (+dox) and noninduced (−dox) cells displayed a concentration-dependent MPP^+^ response with a pEC_50_ value of 4.2 ± 0.1 (Figure 1C). However, dox-induced cells showed a considerably larger maximum effect (E_max_ = 100 ± 2%) as compared to that of noninduced cells (E_max_ = 65 ± 4%), indicating that the MPP^+^-induced cytotoxicity occurred faster and was more pronounced in OCT3-expressing cells (Figure 1C and Figure S1).

Interestingly, a dose-dependent peak response was observed for dox-induced cells in the first 1–2 h after MPP^+^ addition, which reached maximal vehicle-corrected nCI after 40–60 min (Figure 1B,E). Moreover, this peak response could not be observed in noninduced cells, indicating that the response was specific to OCT3-mediated uptake of MPP^+^ (Figure 1A,D). Analysis of the area under the curve (AUC) over the first 60 min resulted in a potency of pEC_50_ = 3.7 ± 0.1 for MPP^+^ on OCT3-expressing cells (Figure 1F).

**Figure 2:**
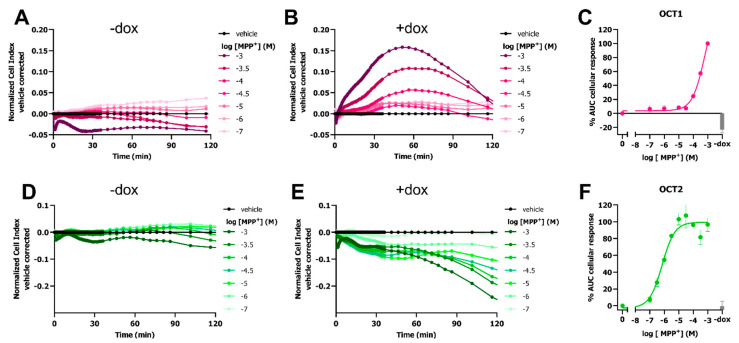
Response of MPP^+^ on HEK293-JumpIn cells with doxycycline-inducible expression of OCT1 or OCT2. Representative vehicle-corrected time-traces of MPP^+^ or vehicle (PBS)-induced changes in cellular impedance on HEK293-JI-OCT1 (**A**,**B**) or OCT2 (**D**,**E**) cells in absence (**A**,**D**) and presence (**B**,**E**) of doxycycline, determined utilizing an xCELLigence RTCA system. Concentration-response curves of MPP^+^ on OCT1 (**C**) and OCT2 (**F**). Gray bar represents 1 mM or 3.16 µM MPP^+^-induced response on noninduced (−dox) OCT1 (**C**) and OCT2 cells (**F**), respectively. AUC cellular response was determined for first 60 (OCT1) or 120 min (OCT2) after cell stimulation with MPP^+^, where 1 mM MPP^+^ response in presence of doxycycline was set at 100%. Data shown are mean ± SEM of at least three independent experiments performed in duplicate.

As transport of MPP^+^ is mediated by various SLCs, it was investigated if MPP^+^-transport could also be observed in HEK-JumpIn cells with inducible OCT1 or OCT2 expression. In line with HEK293-JI-OCT3 cells, no substantial changes in nCI were observed in noninduced (−dox) HEK293-JI-OCT1 and HEK293-JI-OCT2 cells upon stimulation with MPP^+^ (Figure 2A,D). A distinct MPP^+^-induced peak-response in the first 2 h could be observed for dox-induced HEK293-JI-OCT1 cells, similar to OCT3 (Figure 2B). The potency of MPP^+^ for OCT1-mediated uptake was 2.5-fold lower (pEC_50_ = 3.3 ± 0.1) than for that of OCT3 (Figure 2C).

Opposed to OCT1 and OCT3 that displayed an increase in nCI, MPP^+^ addition to dox-treated HEK293-JI-OCT2 cells resulted in a dose-dependent gradual decrease in nCI, which continued to decrease even after 2 h (Figure 2E). In contrast to potencies of MPP^+^ for OCT1 and 3 which were in the high micromolar range, the potency obtained for OCT2-mediated transport of MPP^+^ were 630-fold and 250-fold higher (pEC_50_ = 6.1 ± 0.1) as compared to that of OCT1 and OCT3, respectively (Figure 2F). Taken together, this impedance-based assay elicits a transporter-dependent MPP^+^-induced cellular response for all three OCTs.

### 2.2. OCT Inhibitors Block the MPP^+^-Induced Cellular Response

**Figure 3:**
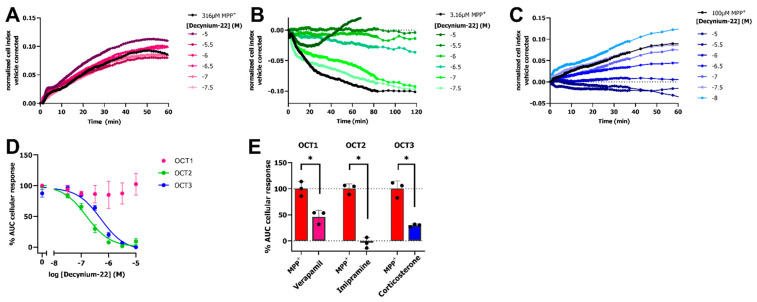
Inhibition of MPP^+^-induced response by decynium-22 or 10 µM of OCT inhibitors in impedance-based transporter assay. Representative vehicle-corrected time-traces of inhibition of MPP^+^ response by decynium-22 on HEK293-JumpIn cells induced with doxycycline to express OCT1 (**A**), OCT2 (**B**), or OCT3 (**C**). Concentration-effect curves of decynium-22 in presence of 316 µM, 100 µM or 3.16 µM MPP^+^ for OCT1, OCT3, and OCT2, respectively (**D**). Inhibition of MPP^+^ response by 10 µM OCT inhibitors verapamil, imipramine, and corticosterone for OCT1, OCT2, and OCT3, respectively(**E**). Statistical significance was determined by a unpaired Student’s *t*-test (* *p* < 0.05). Data shown are mean ± SEM of at least three independent experiments performed in duplicate.

To confirm that OCT-mediated transport of MPP^+^ is the reason for the observed changes in cellular impedance, we investigated if inhibition of the OCTs with the non-selective inhibitor decynium-22 (1-Ethyl-2-[(*E*)-(1-ethylquinolin-2(1*H*)-ylidene)methyl]quinolin-1-ium iodide) abrogates the MPP^+^ response. To this end, dox-induced cells were preincubated with increasing concentrations of decynium-22 for 1 h prior to addition of a submaximal concentration of MPP^+^. Both OCT2 and OCT3 expressing cells that were pretreated with decynium-22 displayed a concentration-dependent reduction in nCI upon stimulation with MPP^+^, while no substantial inhibition was observed after MPP^+^ stimulation on OCT1 expressing cells (Figure 3A–C). Concentration-dependent inhibition of submaximal MPP^+^ response by decynium-22 resulted in potency values of pIC_50_ = 6.8 ± 0.1 and pIC_50_ = 6.4 ± 0.0 for OCT2 and OCT3, respectively (Figure 3D). Unfortunately, pretreatment of cells with decynium-22 did not result in inhibition of OCT1-mediated MPP^+^ transport.

To confirm that inhibition of all three OCTs can be observed utilizing the impedance-based transport assay, dox-induced HEK293-JI-OCT1-3 cells were incubated with 10 µM of the OCT inhibitors verapamil, imipramine, and corticosterone for OCT1, OCT2, and OCT3, respectively. Near complete inhibition was observed for OCT2 using imipramine (remaining OCT2 activity: −3.7 ± 5.9%), while partial inhibition was observed for OCT1 (remaining OCT1 activity: 46 ± 7.3%) and OCT3 (remaining OCT3 activity: 29.8 ± 1.5%) by verapamil and corticosterone, respectively (Figure 3E). Together, this illustrated that OCT activity and inhibition can be determined for all OCT subtypes using the label-free, impedance-based transport assay.

### 2.3. Maintenance of Electrochemical Gradient Is Fundamental for MPP^+^ Response

**Figure 4:**
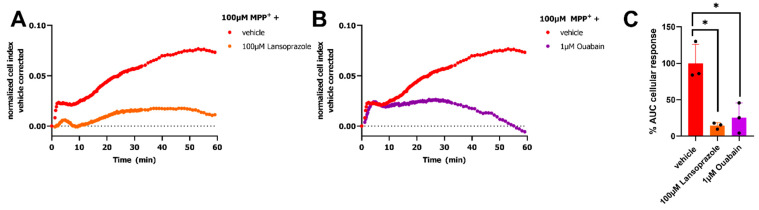
Inhibition of OCT3-mediated MPP^+^ response by H^+^/K^+^-ATPase and Na^+^/K^+^-ATPase inhibitors. Representative time-traces of 100 µM MPP^+^ or vehicle (PBS) -induced response on HEK293-JI-OCT3 cells pretreated for 1 h with either 100 µM lansoprazole (**A**) or 1 µM ouabain (**B**) to inhibit H^+^/K^+^-ATPase or Na^+^/K^+^-ATPase, respectively. Remaining MPP^+^-induced OCT3-activity after pretreatment with ATPase inhibitors (**C**), presented as % AUC cellular response where 100% corresponds to 100 µM MPP^+^ response on vehicle pretreated cells. Statistical significance was determined by an one-way ANOVA with Dunnett’s posthoc test. * *p* < 0.01. Data shown are mean ± SEM of three independent experiments performed in duplicate.

Further pharmacological characterization of the MPP^+^-induced response in our impedance-based transport assay was performed utilizing OCT3 as a prototypical MPP^+^ transporting SLC. As transport of cations like MPP^+^ alters the electrochemical gradient it was investigated if H^+^/K^+^-ATPase and Na^+^/K^+^-ATPase were involved in the MPP^+^-induced peak-response. Dox-induced cells were pretreated for 1 h with either 100 µM of the H^+^/K^+^-ATPase inhibitor lansoprazole or 1 µM of Na^+^/K^+^-ATPase inhibitor ouabain. Pretreatment of the cells with these inhibitors almost fully abrogated the 100 µM MPP^+^ response as shown by a reduced peak-response (Figure 4A–C). This suggested that the ATPases targeted by these inhibitors are fundamental to the observed changes in cellular impedance within the first 2 h after MPP^+^ stimulation, providing some insight in the cellular mechanism involved in this read-out.

### 2.4. Inhibition of OCT3 by Corticosterone Abolishes the MPP^+^-Induced Response

**Figure 5:**
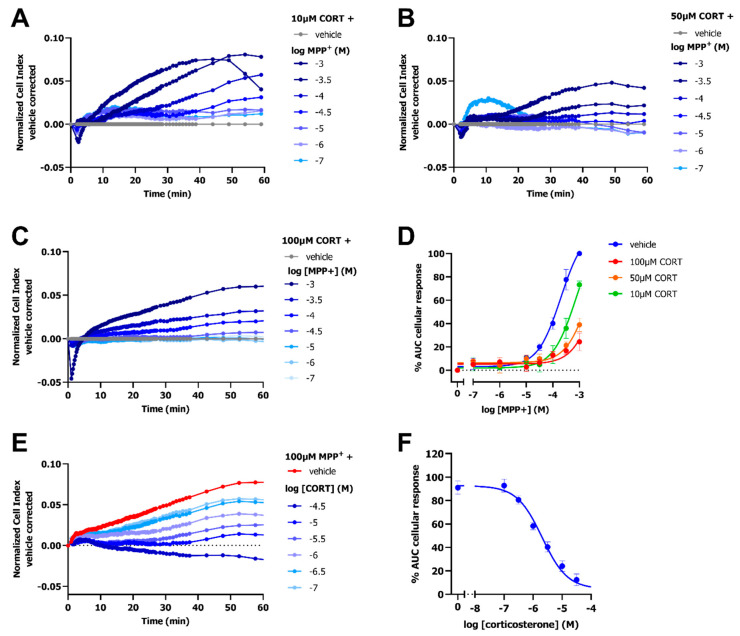
Inhibition of OCT3 by selective inhibitor corticosterone results in a decreased MPP^+^ response in dox-induced HEK293-JI-OCT3 cells. Representative vehicle-corrected time-traces of HEK293-JI-OCT3 cells stimulated with increasing concentration of MPP^+^ after a 1 h pretreatment with either 100 µM (**A**), 50 µM (**B**) or 10 µM corticosterone (CORT) (**C**). Concentration-response curves of MPP^+^ in presence of three different concentrations of corticosterone (**D**). Representative vehicle-corrected time-trace of 100 µM MPP^+^ response after pre-treatment with increasing concentrations of corticosterone (**E**). Dose-dependent inhibition of 100 µM MPP^+^ response by corticosterone (**F**). Data shown are mean ± SEM of at least three independent experiments performed in duplicate.

To further validate the impedance-based transport assay, the inhibitory effect of the selective and competitive OCT3 inhibitor corticosterone was assessed in dox-induced HEK293-JI-OCT3 cells. The dose-dependent MPP^+^-induced peak-response was decreased upon pretreatment with 10 µM, 50 µM, and 100 µM corticosterone, as shown by a reduction in the nCI for all MPP^+^ concentrations (Figure 5A–C). A rightward shift of the MPP^+^ curve was observed in presence of 10 µM corticosterone (pEC_50_ = 3.1 ± 0.1 as compared to pEC_50_ = 3.7 ± 0.1 for vehicle pretreated cells), in line with competitive inhibition at OCT3 (Figure 5D). Moreover, 50 µM and 100 µM corticosterone seemed to cause an even further rightward shift, but EC_50_ values could not be determined due to an almost full abrogation of OCT3-mediated transport of MPP^+^ (Figure 5D). Corticosterone dose-dependently inhibited the 100 µM MPP^+^-induced OCT3 response with a potency of 5.8 ± 0.1 (Figure 5E,F and Table 1), which is comparable to that of literature [23].

### 2.5. Attenuation of OCT3 Activity by TKI-Inhibitors

**Figure 6:**
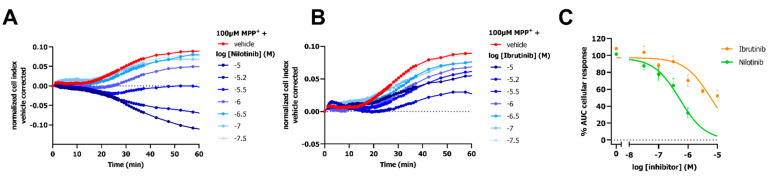
Inhibition of OCT3 by TKI inhibitors detected with an impedance-based transport assay. Representative vehicle and inhibitor corrected time-traces of inhibition of 100 µ M MPP^+^ response by TKI inhibitors nilotinib (**A**) and ibrutinib (**B**) on doxycycline-induced HEK293-JI-OCT3 cells. Concentration-dependent inhibition of 100 µM MPP^+^ response by nilotinib and ibrutinib (**C**). Data shown are mean ± SEM of at least three independent experiments performed in duplicate.

Two TKIs were selected to evaluate the potential of the impedance-based transport assay to identify these structurally diverse compounds as OCT3 inhibitors. OCT3-expressing cells were preincubated with TKIs (i.e., nilotinib and ibrutinib) for 1 h prior to monitoring the MPP^+^- or vehicle-induced cellular response. Cells pretreated with ibrutinib evoked changes in nCI in absence of MPP^+^, while nilotinib did so to a lesser extent (Figure S2C,D). As these effects were long-lasting, the MPP^+^ response in presence of TKIs was corrected for both vehicle and inhibitor-induced effects. Nilotinib and ibrutinib concentration-dependently decreased the 100 µM MPP^+^-induced changes in nCI (Figure 6A–C). Partial inhibition of the MPP^+^ response was observed for ibrutinib with an inhibitory potency of approximately 10 µM (Figure 6C and Table 1). Nilotinib fully inhibited OCT3-Mediated MPP^+^ uptake with a low (sub)micromolar potency (Figure 6C and Table 1).

### 2.6. Inhibitory Potencies from the Impedance-Based Assay Are Comparable to Potencies in an Orthogonal Fluorescent Uptake Assay

**Figure 7:**
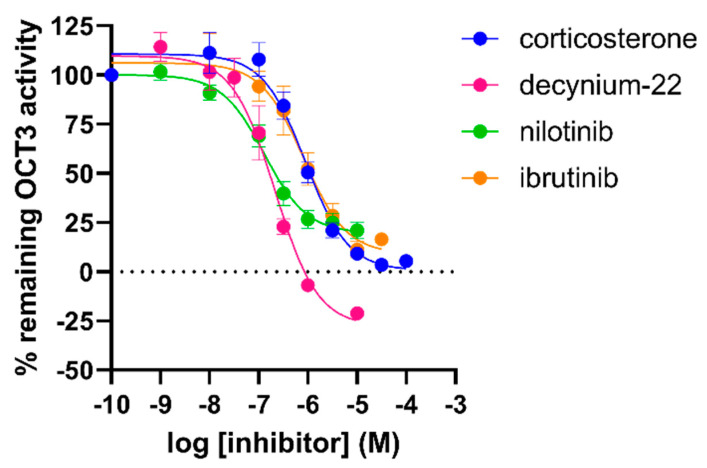
Inhibition of OCT3-mediated uptake of 10 µM DiASP by several inhibitors as measured in doxycycline-induced HEK293-JI-OCT3 cells. DiASP uptake (10 µM) was determined after 1 h incubation on cells pretreated for 10 min with increasing concentrations of inhibitor. Data shown are mean ± SEM of at least three independent experiments performed in triplicate.

Next, to further validate the inhibitory potencies obtained in this new assay, the OCT3 inhibitors were tested in an orthogonal uptake assay using the fluorescent MPP^+^ analogue DiASP (4-(4-diethylaminostyryl)-1-methyl-pyridinium iodide). Dox-induced HEK293-JI-OCT3 cells were preincubated for 10 min with inhibitor and uptake of 10 µM DiASP was quantified after 1 h. Inhibitory potencies of reference OCT3 inhibitors corticosterone and decynium-22 obtained from DiASP uptake assay were similar to potencies derived from the impedance-based transport assay (Figure 7 and Table 1). In contrast, inhibitory potencies of TKIs were 4- to 10-fold lower in competition with 100 µM MPP^+^ as compared to that of 10 µM DiASP in the impedance-based assay and fluorescent uptake assay, respectively (Figure 7 and Table 1).

### 2.7. Detection of OCT3-Mediated Transport of MPP^+^ and Inhibition by Corticosterone in Hela Cells

**Figure 8:**
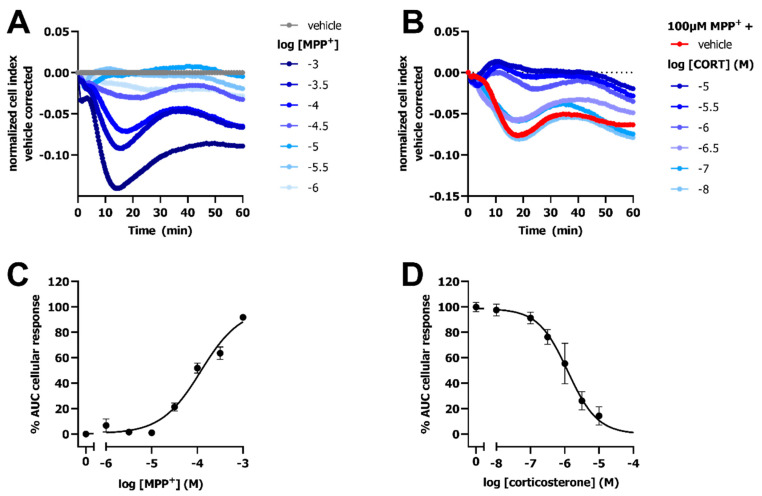
OCT3-mediated transport of MPP^+^ and MPP^+^-inhibition by corticosterone on HeLa cells endogenously expressing OCT3. Representative time trace of MPP^+^-induced changes in CI on HeLa cells endogenously expressing OCT3 (**A**). Representative time trace of inhibition of 100 µM MPP^+^ uptake in HeLa cells pretreated for 1 h with increasing concentrations of corticosterone (**B**). Concentration-response curve of MPP^+^ on HeLa cells (**C**), where 1 mM MPP^+^ response was set at 100%. Concentration-inhibition curve of corticosterone in presence of 100 µM MPP^+^ (**D**), which was set at 100%, while response of vehicle-pretreated cells was set at 0%. Data shown are mean ± SEM of at least three independent experiments performed in duplicate.

Next, the impedance-based transport assay was evaluated for its ability to detect OCT-mediated transport of MPP^+^ on cells endogenously expressing the transporter. Here, HeLa cells were utilized as a model cell-line with endogenous OCT3 expression [1,24]. Stimulation of HeLa cells with increasing MPP^+^ concentrations resulted in a dose-dependent decrease in vehicle-corrected nCI up to 14–18 min followed by a gradual increase in impedance (Figure 8A). The 100 µM MPP^+^-induced response could be concentration-dependently inhibited by corticosterone (Figure 8B). Potencies of MPP^+^ (pEC_50_ = 3.9 ± 0.1) and corticosterone (pIC_50_ = 6.0 ± 0.1) obtained in the impedance-based transport assay using HeLa cells were in line with HEK293-JI-OCT3 cells (Figure 1 and Figure 8), indicating that the observed response is mediated via OCT3. Taken together, the label-free impedance-based transport assay can measure OCT3 activity and inhibition on both overexpressing and endogenous OCT3 expressing cells.

## 3. Discussion

Organic cation transporters are important targets, as they transport the widely used antidiabetic drug metformin and are prone to DDIs due to their pivotal role in drug absorption, disposition, and excretion. However, on some occasions these DDIs might be beneficial to prevent localized toxicity, e.g., nephrotoxicity of chemotherapeutic agents can be prevented by coadministration of OCT2 inhibitors [25] and OCT3 inhibitors were shown to counteract cardiac toxicity [9,12]. In addition, selective targeting of OCTs is currently explored as a strategy to potentiate antidepressant effects [26,27,28]. Unfortunately, there are not that many OCT inhibitors, and they tend to have low selectivity. Current methodologies to screen for OCT inhibition, and actually that of SLCs in general, is rather limited [29], and despite their various limitations, radioligand-based uptake and binding assays are still the principal method to investigate inhibition of OCTs [7,16]. Although fluorescence-based assays are a nonradioactive alternative with potential for high-throughput screening [30], these assays still require a labelled substrate, which is not always available, and are prone to artifacts due to auto-fluorescence or quenching capacity of the tested compound [16]. Moreover, both assay types are generally “end point” assays, such that time-dependency cannot be easily studied in real-time. Recently label-free techniques, as a new technology, were introduced to study transporter activity [16,19,31,32]. We previously introduced the so-called TRACT assay requiring a GPCR partner to detect SLC-transport activity via a mutual substrate/agonist [17,18,19]. Endogenously, SLCs and GPCRs are not always coexpressed, and thus would require transient expression of either the SLC or GPCR. In this study, we developed a novel label-free impedance-based assay that can detect transport of a substrate directly, i.e., without the need to coexpress a GPCR. Specifically, with this assay OCT1-3 transport activity is detected utilizing the cellular response evoked by uptake of the neurotoxic substrate MPP^+^.

MPP^+^ induces changes in cellular impedance upon transport into the cell by all three OCTs (Figure 1 and Figure 2). Advantageously, OCT1-3-mediated uptake of MPP^+^ could already be detected within the first hour after MPP^+^ addition, making this assay format more suitable for screening applications, as conventional cytotoxicity assays require measurement of cell viability over 24–48 h [33]. Moreover, label-free impedance-based cytotoxic analysis of MPP^+^ on HEK293-JI-OCT3 cells had a relatively low sensitivity compared to the response observed in the first hours, as vehicle and dox-treated cells both displayed reduced cell viability over 52 h period with identical potencies and only a modest (35%) difference in E_max_ (Figure 1A–C). The observed MPP^+^-induced changes in cellular impedance in the first 1–2 h, were a direct consequence of its transport by OCTs as no response was detected in cells lacking OCT expression. Potencies for MPP^+^ uptake by OCT2 and OCT3 were in a similar range as reported affinity values from an electrophysiology and uptake assay, respectively [34,35], while OCT1 displayed an over 10-fold lower potency as compared to its affinity for OCT1 in a radioligand-uptake assay [2,5,36]. Of note, potencies obtained from the functional impedance-based transport assay are not directly comparable to previously published K_m_ values, as the potency not only depends on a substrate’s affinity for the transporter but is also influenced by the rate of transport and the cellular response evoked by MPP^+^. As the response is solely dependent on MPP^+^ uptake the presented assay format could facilitate the study of other MPP^+^ transporting SLCs, such as DAT and NET [37].

Even though the complex mechanism of MPP^+^-induced toxicity and especially the events that occur in the first hours are still not fully understood, we hypothesize that the observed MPP^+^ peak response in the impedance-based transport assay is a consequence of dysregulation of mitochondrial membrane potential and oxidative stress that results in alterations in cell morphology [38,39]. MPP^+^ was shown to inhibit mitochondrial complex I and rapidly forms reactive oxygen species (ROS) in the early phase eventually leading to depletion of intracellular ATP pools and, induction of apoptosis, resulting in cell shrinkage and cell death [40,41,42]. Oxidative stress induced by MPP^+^ is known to disrupt microtubules and causes cytoskeletal rearrangements that alter cell morphology, a process that is already apparent in the first hour after MPP^+^ treatment [39,40,43,44]. Indeed, these cytoskeletal rearrangements can be detected in impedance-based assays and might reflect the initial peak response, while the subsequent decrease in cellular impedance may arise from induction of apoptosis. Besides, exposure of cells to MPP^+^ was shown to reduce Na^+^/K^+^-ATPase activity due to reduced energy metabolism, and thereby hampers OCT-mediated uptake of cations [45,46]. This is in line with abrogation of the MPP^+^ response in cells pretreated with ouabain and lansoprazole, a Na^+^/K^+^-ATPase and H^+^/K^+^-ATPase inhibitor, respectively (Figure 3).

Interestingly, the MPP^+^ response observed on HEK293-JI-OCT2 cells displayed a gradual decrease in nCI, due to a lack of the initial peak response observed for both OCT1 and OCT3 expressing cells. A potential reason for this might be that MPP^+^ has a higher potency for OCT2, which may result in a more rapid accumulation of MPP^+^ in the cells and thus a more rapid onset of toxic effects leading to an immediate decrease in the nCI. It remains to be investigated why OCT2-mediated uptake induces a response distinct from that observed for OCT1 and OCT3.

Next, various OCT-inhibitors were evaluated in the impedance-based transport assay. Competitive inhibition of OCTs fully abrogated the MPP^+^ response with inhibitory potencies of decynium-22 for OCT2 and OCT3 that were comparable to those that were reported in an [^3^H]MPP^+^ uptake assay [3,47]. However, the high MPP^+^ concentration needed to detect an OCT1-mediated response limited assessment of inhibitors with high micromolar potencies as seen for decynium-22, while it was possible to detect partial inhibition by the known potent OCT1 inhibitor verapamil (Figure 3) [7,48]. For detection of high-affinity inhibitors, it was advised to use a low substrate concentration as influence of probe concentration on inhibitory potency was recently uncovered when using high concentrations MPP^+^ [15,49]. Therefore, we selected the lowest MPP^+^ concentration providing a robust assay window (i.e., 316 µM for OCT1, 3.16 µM for OCT2 and 100 µM for OCT3) to assess inhibitor potencies. The observed rank order of tested OCT3 inhibitors in the impedance-based transport assay was similar to the values obtained in the orthogonal DiASP uptake assay. Moreover, potencies of corticosterone and decynium-22 in both assay formats were comparable to potencies reported in literature for a radiolabeled MPP^+^ uptake assay [47]. Although the potencies of TKIs for OCT3-inhibition were considerably lower in our impedance-based assay as compared to that of the fluorescent uptake assay (Table 1), the inhibitory potency for nilotinib was in agreement with its potency to inhibit human OCT3-mediated metformin uptake [14]. Indeed, consistently lower potencies were reported for OCT inhibitors utilizing MPP^+^ as a substrate compared to that of its fluorescent analogue (Di)ASP due to probe dependency [15,50]. Of note, the potencies of TKIs in our assay might additionally be influenced by TKI-induced changes in cell morphology. Thus, OCT and TKI inhibitors could be evaluated using the impedance-based transport assay, though an “inhibitor only”-control is required to exclude inhibitor-induced effects (Figure S2).

The MPP^+^ response on HeLa cells display an initial decrease in contrast to HEK cells that displays an initial increase. As shown previously, the observed cellular impedance is highly dependent on the cellular background, where different even opposing effects can be seen when a compound is tested in different cell-lines [51,52,53]. Potencies for MPP^+^ transport and inhibition by corticosterone were similar for both overexpressing HEK293-JI-OCT3 cells and HeLa cells endogenously expressing OCT3. Thus, this impedance-based transport assay might be a valuable tool for assessment of OCT inhibition on clinically relevant cells. The ability to detect inhibition of MPP^+^ transport on cells endogenously expressing the transporter is promising, as it makes construction of artificial, overexpressing cell lines redundant. Besides, various polyspecific cation-transporting SLCs (i.e., OCTs, MATEs etc.) are generally coexpressed on cells [54,55]. Thus, the presented assay has the potential to investigate several transporters at once, and screening multiple targets could potentially lead to a better prediction of the in vivo inhibitory effect of drug candidates prior to their assessment in clinical studies.

Since many elderly patients are these days treated with multiple drugs for various conditions, prior knowledge of potential DDIs is of utmost importance. The ability to measure OCT inhibition in real-time using the impedance-based transport assay makes it an attractive alternative for screening of drug candidates for interaction with OCTs in the early phases of drug discovery, as compared to the low-throughput [^3^H]MPP^+^ uptake assay that is recommended by the FDA and EMA. Moreover, screening of drug candidates on clinically relevant cell-lines instead of cells overexpressing the transporter could provide a more physiologically relevant setting than conventional uptake assays.

In conclusion, we developed a label-free, impedance-based transport assay that is able to detect OCT-mediated uptake of MPP^+^ in heterologously and endogenously expressing cells, enabling a quantitative analysis of transport inhibition. The real-time assay format combined with the fast detection of the MPP^+^ response make this assay a valuable tool for screening applications, such as early identification of clinical candidates interacting with OCTs and potentially provoking DDIs. Hence, this novel assay could aid in the screening and further development of selective OCT inhibitors and inhibitors of other SLCs that transport MPP^+^.

## 4. Materials and Methods

### 4.1. Materials

Jump In T-Rex human embryonic kidney 293 cells (HEK293-JumpIn) with doxycycline-inducible expression of human OCT1, OCT2 or OCT3 (HEK293-JI-OCT1-3) were provided by Research Center for Molecular Medicine, Medical University of Vienna, Austria (CEMM) and constructed as reported previously [17]. HeLa cells were obtained from AddexBio (C0008001, San Diego, CA, USA). Corticosterone, 1-Ethyl-2-[(1-ethyl-2(1*H*)-quinolinylidene)methyl]quinolinium iodide (decynium-22), 1-Methyl-4-phenylpyridinium iodide (MPP^+^), 4-(4-diethylaminostyryl)-1-methyl-pyridinium iodide (DiASP), doxycycline hyclate, lansoprazole, ouabain, Dulbecco’s modified Eagles medium (DMEM), Minimum Essential Medium Eagle (MEM) and G418 were purchased from Sigma Aldrich (Merck, Darmstadt, Germany). Nilotinib, and ibrutinib were purchased from MedChemExpress (South Brunswick, NJ, USA). Hank’s Balanced Salt Solution (HBSS) and poly-D-lysine were obtained from ThermoFisher Scientific (Waltham, MA, USA). All other chemicals were bought from standard commercial resources and were of analytical grade.

### 4.2. Cell Culture

HEK293-JI-OCT1-3 cells were cultured in DMEM supplemented with 2 mM Glutamax, 10% fetal calf serum (FCS), 100 µg/mL streptomycin and 100 IU/mL penicillin. Prior to experiments cells were cultured for two passages in selection medium, i.e., culture medium supplemented with 2 mg/mL G418 and 5 µg/mL blasticidine, to select for OCT1-3 positive cells. Prior to use in functional experiments, cells were grown for at least 24 h in regular culture medium and were cultured to reach approximately 80% confluency. HeLa cells were cultured in MEM supplemented with 10% FCS, 2 mM Glutamax, 100 µg/mL streptomycin and 100 IU/mL penicillin.

### 4.3. Impedance-Based Transport Assay (xCELLigence)

Label-free cellular impedance measurements were performed using an xCELLigence Real-Time Cell Analyzer (RTCA) SP system (ACEA biosciences, San Diego, CA, USA) as described previously [17,18]. In short, the xCELLigence biosensor detects changes in cell morphology, adhesion, and proliferation through impedance of the electron flow. Baseline impedance of the E-plate was determined in 40 µL culture medium with or without 1 µg/mL dox to induce OCT expression. HEK293-JI-OCT1-3 (60,000 cells/well) or HeLa cells (20,000 cells/well) were seeded in 50 µL culture medium and cells were allowed to settle in the E-plate for 30 min at RT. Next, the E-plate was placed in the xCELLigence station at 37 °C, 5% CO_2,_ and cell growth was monitored every 15 min for 22 h to reach confluency. Hereafter, cells were treated with 5 µL inhibitor at increasing concentrations (ranging from 10^−4.5^ M to 10^−7^ M for corticosterone, and 10^−5^ M to 10^−7.5^ M for nilotinib, ibrutinib and, 10^−5^ M to 10^−8^ M for decynium-22) or vehicle (i.e., PBS supplemented with 0.1% DMSO), which were added to the E-plate utilizing a VIAFLO 96 handheld electronic 96 channel pipette (Integra Bioscience, Tokyo, Japan). Inhibitor responses were monitored for 1 h at 1 min intervals. Transporter activity was subsequently induced by addition of 5 µL pre-warmed MPP^+^ (substrate) or PBS (vehicle) using the VIAFLO 96. For MPP^+^ dose-response curves cells were stimulated with MPP^+^ concentrations ranging from 10^−3^M to 10^−7^ M. For inhibitor studies cells were stimulated with a submaximal concentration of MPP^+^, which corresponds to 100 µM for OCT3, 316 µM for OCT1, and 3.16 µM for OCT2. Changes in cell morphology were monitored every 15 s for 30 min, followed by every minute for 30 min and finally every 5 min for 1 h. For cytotoxicity experiments, this measurement schedule was extended by monitoring every 15 min up to a total of 52 h.

### 4.4. Fluorescent Substrate Uptake Assay

Fluorescent uptake assays with DiASP were performed as described previously [56]. In brief, HEK293-JumpIn-OCT3 cells were seeded (60,000 cells/well) in medium containing 1 µg/mL doxycycline on a poly-D-lysine-coated white walled, clear bottom 96-well plate 24 h prior to the fluorescent uptake measurement. On the next day, cells were washed with 200 µL prewarmed HBSS and cells were incubated with 100 µL inhibitor (concentrations ranging from 10^−5^ M to 10^−8^ M) in HBSS for 10 min at 37 °C. The uptake of fluorescent DiASP was initiated by the addition of 100 µL uptake mix consisting of 10 µM DiASP in HBSS supplemented with 50 µM trypan blue to quench the extracellularly present DiASP. DiASP uptake was quantified after 1 h at 37 °C by measuring fluorescence at excitation wavelength/bandwidth 475–14 nm and emission wavelength/bandwidth 609–14 nm utilizing a Flexstation 3 Multi-mode microplate reader (Molecular devices, Workingham, UK).

### 4.5. Data Analysis

All data were analyzed using Graphpad prism 9.0 (Graphpad software Inc., San Diego, CA, USA). Data are presented as mean ± SEM of at least three independent experiments performed in duplicate, unless stated otherwise.

For xCELLigence experiments, RTCA software v2.0 (ACEA Biosciences, San Diego, CA, USA) was used to normalize the CI to the time-point prior to substrate addition. Next, to correct for substrate-independent effects the vehicle response was removed from all data points. For inhibitor experiments, data were also corrected for inhibitor-induced effects when the inhibitor produced a substantial cellular response in absence of MPP^+^. Vehicle and inhibitor corrected data was analyzed by calculating the net AUC over the first 60 min for OCT1 and OCT3 or 120 min for OCT2 to reach a maximal peak response for all concentrations after stimulation with MPP^+^. The 1 mM MPP^+^-induced response (AUC) in presence of dox was taken as the maximum response (E_max_) and set to 100%. Notably, conditions in which the MPP^+^ response in presence of inhibitor after correction for vehicle and inhibitor-induced effects obtained a negative net AUC were omitted from analyses as this could indicate inhibitor-induced toxic effects, as observed for Nilotinib. Concentration-response curves were fitted using a non-linear regression three parameter response model to obtain pEC_50_ and pIC_50_ values.

For fluorescent uptake assays, data were normalized for background uptake of DiASP by subtracting the baseline-fluorescence directly after addition of uptake mix. Data was converted to percentage OCT3 activity whereby the fluorescent DiASP signal in absence of inhibitors was set as 100% and fluorescence in presence of 100 µM corticosterone was considered as total inhibition (0%). Concentration-response curves were fitted using a non-linear regression three-parameter response model to obtain pIC_50_ values.

Statistical analyses were also performed using Graphpad Prism 9.0. Significant difference between vehicle and inhibitor-induced response was determined using an unpaired Student’s *t*-test. Significant difference between the mean MPP^+^ response in vehicle and ATPase inhibitor pretreated cells was determined using a one-way ANOVA with Dunnett’s posthoc test. Potency values of impedance-based transport assay and fluorescent uptake assay were compared using unpaired Student’s *t*-test with multiple comparison using the Holm–Sidak method.

## Figures and Tables

**Table 1:** Potency of OCT3 and TKI inhibitors to inhibit OCT3 activity in dox-induced HEK293-JI-OCT3 cells. Data shown are mean ± SEM of (n) experiments performed in duplicate or triplicate for impedance-based transport (xCELLigence) assay and fluorescent uptake assay, respectively.

Compound	pIC_50_	
	xCELLigence	Fluorescent Uptake Assay
Decynium-22	6.4 ± 0.0 (3)	6.7 ± 0.1 (3)
Corticosterone	5.8 ± 0.1 (7)	6.1 ± 0.1 (6)
Nilotinib	6.3 ± 0.1 (3) *	6.9 ± 0.1 (3)
Ibrutinib	5.1 ± 0.2 (3) ***	6.1 ± 0.2 (4)

An unpaired Student’s *t*-test with multiple comparison using the Holm–Sidak method * *p* < 0.05, *** *p* < 0.001.

## Data Availability

The data presented in this article are available from the corresponding author on reasonable request.

## Supplementary Materials

The following are available online at www.mdpi.com/article/10.3390/ijms23031203/s1.

## Abbreviations

AUC	Area under the curve
DAT	Dopamine transporter
DDIs	Drug-drug interactions
DiASP	4-(4-diethylaminostyryl)-1-methyl-pyridinium iodide
dox	Doxycycline
ENT1	Equilibrative nucleoside transporter 1
E_max_	Maximum response
GPCR	G protein-coupled receptor
HEK	Human embryonic kidney
MATE	Multidrug and toxic extrusion
MPP^+^	1-Methyl-4-phenylpyridinium iodide
NET	Norepinephrine transporter
OCT	Organic cation transporter
pEC_50_	−log (EC_50_) where EC_50_ is the concentration required to obtain half maximal response by a compound
pIC_50_	−log (IC50) where IC50 is concentration inhibitor required to reduce the response by 50%
ROS	Reactive oxygen species
SLC	Solute carrier protein
TKI	Tyrosine kinase inhibitor
TRACT	Transport activity through receptor activation
