# Supplementary material for: MPP+-Induced Changes in Cellular Impedance as a Measure for Organic Cation Transporter (SLC22A1-3) Activity and Inhibition
Source: Int J Mol Sci. 2022 Jan 21;23(3):1203. doi: 10.3390/ijms23031203 (PMC8835585; doi:10.3390/ijms23031203)
Supplement: Supplementary file 1 [file ijms-23-01203-s001.zip › ijms-1540985-supplementary.pdf]

Supplementary information

MPP$^+$-induced changes in cellular impedance as a measure for Organic Cation Transporter (SLC22A1-3) activity and inhibition

T.A.M. Mocking[1], H.J. Sijben[1], Y.W. Vermeulen[1], A.P. IJzerman[1], L.H. Heitman[1,2,*]

[1]Division of Drug Discovery and Safety, Leiden Academic Centre for Drug Research, Leiden University, the Netherlands.
[2]Oncode Institute, Leiden, The Netherlands.
[*] corresponding author, l.h.heitman@lacdr.leidenuniv.nl

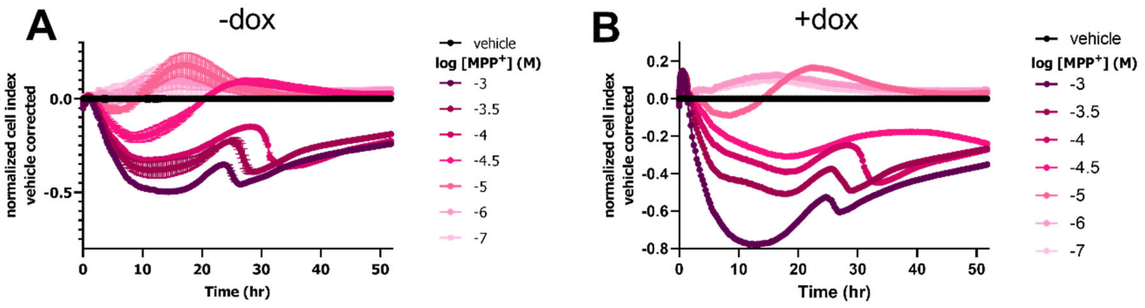

Figure S1. Vehicle-corrected MPP$^+$ response on HEK293-JI-OCT3 cells. Representative vehicle-corrected time-traces of MPP$^+$ response in absence (A) and presence (B) of dox-induced OCT3 expression as measured by the RTCA xCELLigence system for 52 hr. Data shown as mean ± SD of a representative experiment of at least 3 experiments performed in duplicate.

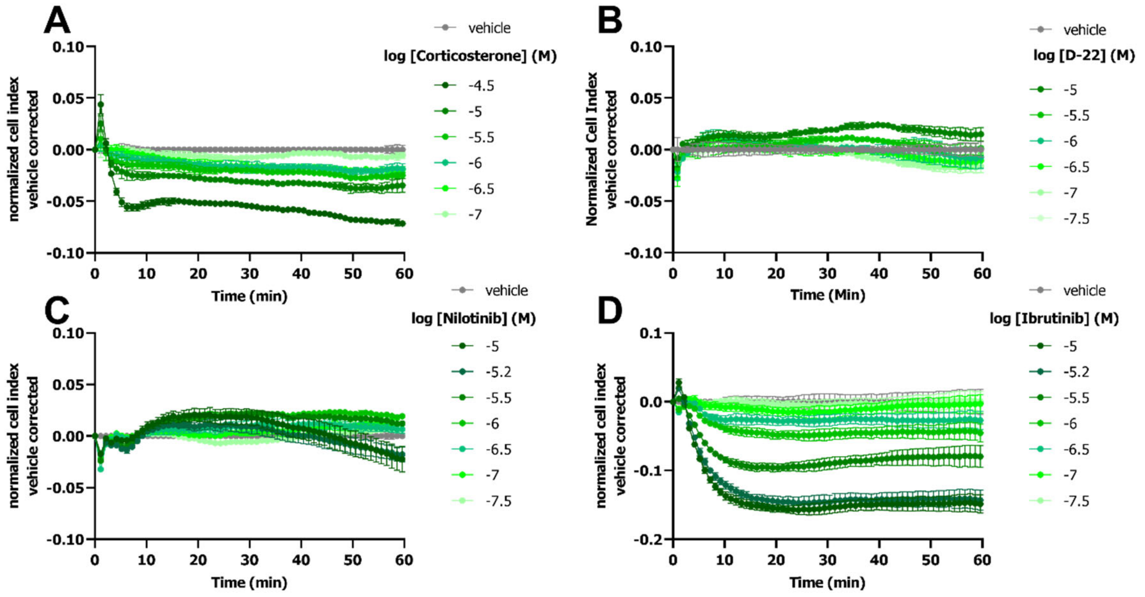

Figure S2. Inhibitor-induced cellular response in HEK293-JumpIn-OCT3 cells. Representative vehicle-corrected time traces of cells pretreated with increasing concentrations of Corticosterone (A), Decynium-22 (D-22) (B), Nilotinib (C) or Ibrutinib (D). Data shown as mean ± SD of a representative experiment of at least 3 experiments performed in duplicate.
